# MeCP2 binds to 5hmc enriched within active genes and accessible chromatin in the nervous system

**DOI:** 10.1186/1756-8935-6-S1-P52

**Published:** 2013-03-18

**Authors:** Marian Mellen, Pinar Ayata, Scott Dewell, Skirmantas Kriaucionis, Nathaniel Heintz

**Affiliations:** 1 Laboratory of Molecular Biology, Howard Hughes Medical Institute, The Rockefeller University, 1230 York Avenue, New York, NY 10065, USA; 2 Genomics Resource Center, The Rockefeller University, 1230 York Avenue, New York, NY 10065, USA; 3 Laboratory of Epigenetic Mechanisms, Ludwig Institute for Cancer Research, The University of Oxford, ORCRB, Oxford, OX37DQ, UK

## 

The very high levels of 5-hydroxymethylcytosine (5hmC) present in neuronal genomes and its accumulation within gene bodies suggests that the epigenetic mechanisms interpreting 5hmC in the central nervous system (CNS) may differ from those present in embryonic stem (ES) cells. Here we present the first quantitative, genome-wide analysis of 5hmC, 5-methylcytosine (5mC) and gene expression in identified, terminally differentiated CNS cell types *in vivo*. We report that the high level of 5hmC present in neurons is enriched in active transcription units, and that surprisingly strong depletion of 5mC over gene bodies is observed for these genes. However, the relative contribution of these epigenetic marks to gene expression depends critically on cell type. We identify methyl-CpG binding protein 2 (MeCP2) as the major 5hmC binding protein in the brain, and demonstrate that MeCP2 binds 5hmC and 5mC containing DNA with similar high affinities. The Rett Syndrome causing mutation of residue R133C in the MeCP2 methyl-CpG binding domain (MBD) preferentially inhibit 5hmC binding. Loss of MeCP2 does not alter the genomic distribution of 5hmC. These findings demonstrate that 5hmC and MeCP2 constitute a novel, cell specific epigenetic mechanism for regulation of chromatin structure and gene expression in the mammalian nervous system, and they provide new mechanistic insights into the pathophysiology of Rett Syndrome (RTT).

